# Synergy of Nb Doping and Surface Alloy Enhanced on Water–Alkali Electrocatalytic Hydrogen Generation Performance in Ti‐Based MXene

**DOI:** 10.1002/advs.201900116

**Published:** 2019-04-05

**Authors:** Cheng‐Feng Du, Xiaoli Sun, Hong Yu, Qinghua Liang, Khang Ngoc Dinh, Yun Zheng, Yubo Luo, Zhiguo Wang, Qingyu Yan

**Affiliations:** ^1^ State Key Laboratory of Solidification Processing Center of Advanced Lubrication and Seal Materials Northwestern Polytechnical University Xi'an Shaanxi 710072 P. R. China; ^2^ School of Materials Science and Engineering Nanyang Technological University 50 Nanyang Avenue 639798 Singapore Singapore; ^3^ School of Electronics Science and Engineering University of Electronic Science and Technology of China Chengdu 610054 P. R. China

**Keywords:** DFT calculation, doping, hydrogen evolution reaction, MXene, surface alloying modification

## Abstract

Presented are the theoretical calculation and experimental studies of a Ti_3_C_2_T*_x_* MXene‐based nanohybrid with simultaneous Nb doping and surface transition metal alloy modification. Guided by the density functional theory calculation, the Nb doping can move up the Fermi energy level to the conduction band, thus enhancing the electronic conductivity. Meanwhile, the surface modification by Ni/Co alloy can moderate the surface M–H affinity, which will further enhance the hydrogen evolution reaction (HER) activity. A series of Ni/Co alloy attached on Nb‐doped Ti_3_C_2_T*_x_* MXene nanohybrids (denoted as NiCo@NTM) are successfully prepared. As expected, the Ni_0.9_Co_0.1_@ NTM nanohybrids present an extraordinary HER activity in alkaline solution, which only needs an overpotential (η) of 43.4 mV to reach the current density of 10 mA cm^−2^ in 1 m KOH solution and shows good stability. The performance of the Ni_0.9_Co_0.1_@ NTM nanohybrids is comparable to the commercial 10% Pt/C electrode (34.4 mV@10 mA cm^−2^) and is better than most state‐of‐the‐art Pt‐free HER catalysts. Inspired by the facile synthesis process and chemical versatility of both MXene and transition metal alloys, the nanohybrids reported here are promising non‐noble metal electrocatalysts for water–alkali electrolysis.

With the increasing global environmental concerns and the requirement of social energy consumption, the industrial scale production of renewable clean energy has become an imminent demand in modern society. Due to the high energy density and the completely environment‐friendly end products, hydrogen was considered as one of the most promising candidates for future energy carrier.^1^ Among various hydrogen evolution reaction (HER) approaches, the electrocatalytic route from water is still one of the most cost‐efficient and productive choice for future industrial‐scale hydrogen production.^2^ However, nowadays the best HER electrocatalyst with high activity and fast kinetics is still highly relied on the expensive noble metal catalysts such as Pt, which limits its industrial‐scale application.^3^, ^4^, ^5^ Therefore, the exploration of Earth‐abundant HER catalyst with comparable catalytic activity and kinetics to Pt has become the most urgent task for the future hydrogen economy.

Generally, the HER activity of a catalyst in alkaline solution might be dominated by two steps: the formation of an adsorbed hydrogen atom on catalyst surface (or known as Volmer step) and the combination/release of molecular hydrogen from catalyst surface (Heyrovsky or Tafel step).^3^, ^6^ From the thermodynamic point of view, both steps are closely related to the bonding strength between the catalyst and hydrogen‐containing intermediate products (Cat—H).^7^ As for Pt, a moderate strength of Pt—H bonding endows its excellent HER activity.^8^ Therefore, intensive efforts have been made on the development of binary or ternary HER catalysts with moderate Cat—H bonding strength.

2D MXene materials with enlarged surface area and fast carrier mobility recently have drawn intensive attention in the field of electrocatalysis.^5^, ^9^, ^10^ Notably, the most studied Ti_3_C_2_T*_x_* MXene usually presents poor electrocatalytic HER activity because of the strong surface Ti—H bonding strength. Therefore, efforts have been made on the surface alloying modification (SAM) of MXene materials based on their abundant surface exposed metal sites. Recently, the Nb_2_CT*_x_* MXene with highly reducible surface has been applied as a functional substrate for the Pt–SAM nanohybrids, which presents the enhanced water–gas shift reaction activity.^10^ Meanwhile, the single Pt atoms surface‐anchored Mo_2_TiC_2_T*_x_* also demonstrated a good HER catalyst.^5^ However, research on SAM of MXene was still at an early stage, in consideration of the high cost of Pt, low loading mass of single atoms catalyst, and the complicated synthesis progress; a more cost‐efficient and easily available MXene‐based electrocatalyst is still desired toward the practical applications.

Herein, the reducible Nb species and inexpensive Ni/Co alloy were selected as a dopant and SAM reagent toward the novel Ti_3_C_2_T*_x_* MXene‐based HER electrocatalyst, respectively. The theoretical calculation and experimental studies on the synergy of Nb doping and Ni/Co‐SAM were performed. As a result, the surface Ni/Co alloy modified Nb‐doped Ti_3_C_2_T*_x_* MXene nanohybrids (denoted as NiCo@NTM) have shown the enhancement on water–alkali electrocatalytic HER activity. Especially, the overpotential (η) of 43.4 mV to reach the current density of 10 mA cm^−2^ in 1 m KOH solution for Ni_0.9_Co_0.1_@NTM presents a comparable performance to the commercial 10% Pt/C electrode (34.4 mV@10 mA cm^−2^) and better than most of the state‐of‐the‐art Pt‐free HER catalysts.

In order to explore the intrinsic properties variation and determine the active site after doping and SAM, the theoretical calculation base on density functional theory (DFT) was conducted first (details of simulation method can be seen in the Supporting Information). The total density of states (DOS) and projected density of states (PDOS) of the six MXene monolayers (Ti_3_C_2_O_2_, Nb‐doped Ti_3_C_2_O_2_, Ti_3_C_2_F_2_, Nb‐doped Ti_3_C_2_F_2_, Ti_3_C_2_(OH)_2_, and Nb‐doped Ti_3_C_2_(OH)_2_) were drawn in Figure S1 in the Supporting Information. As a result, all the six monolayers show the similar conductor property, in which the top of valence band and bottom of conduction band are mainly composed of Ti 3d state. After Nb doping, which replaces the surface Ti atom, and there are Nb 4d state peaks that appears nearby the Fermi energy level, making the Fermi energy level move up to the conduction band thus improving the conductivity.

According to thermodynamics, the hydrogen adsorption Gibbs free energy (Δ*G*_H_) could be a common criterion for evaluating the HER activity. For an optimal catalyst, the Δ*G*_H_ should be close to zero.^11^ Therefore, the reactive hydrogen atom (H*) adsorbed structures of Ti_3_C_2_ and Nb‐doped Ti_3_C_2_ with three surface terminated groups were first taken into consideration, respectively (**Figure** **1**a–b; Figure S2, Supporting Information). From the calculations, it could be found that the Δ*G*_H_ for the H* adsorbed on the terminated —O group above Ti atoms is −0.25 eV. After doping with Nb atom, the calculated Δ*G*_H_ values above the O site nearby Nb atom and away from Nb atom are −0.14 and −0.23 eV, respectively, indicating that the enhanced HER performance can be attributed to Nb doping. A similar trend also observed on —OH and —F terminated Ti_3_C_2_ and Nb‐doped Ti_3_C_2_ monolayers (Figure S2, Supporting Information). However, the calculated Δ*G*_H_ for Ti_3_C_2_(OH)_2_, Nb‐doped Ti_3_C_2_(OH)_2_, Ti_3_C_2_F_2_, and Nb‐doped Ti_3_C_2_F_2_ are −0.70, −0.65, −0.94, and −0.70 eV, respectively, which are all higher than the −O terminated structure, indicating the surface terminated −O might be the preferable H* adsorption site (Figure S2, Supporting Information). The calculated results are in accordance with previous DFT studies,^12^ hence the Δ*G*_H_ based on —O terminated structures are taken into consideration. In addition, although the Nb doping can reduce the Δ*G*_H_ of pristine Ti_3_C_2_T*_x_* MXene, the remission of strong surface H* affinity by Nb doping is still not enough for the requirement of advanced HER catalyst. As we all know, the compounds containing Ni and/or Co usually shows enhanced HER performance. Therefore, the Nb‐doped Ti_3_C_2_T*_x_* monolayers with Co/Ni atoms on their surface were also evaluated. As shown in Figure 1c, when Co/Ni replace the surface Ti atom, three O sites for H* adsorption were tested: 1) the O_1_ site is nearby Nb atom but far away from Co/Ni atom; 2) the O_2_ site is nearby both Nb and Co/Ni atoms; 3) the O_3_ site is far away from both Nb and Co/Ni atoms. As a result, the Δ*G*_H_ of H* on O_1_ to O_3_ sites are −0.08 eV, −0.25 eV, and −0.17 eV for Co–Nb‐doped Ti_3_C_2_O_2_ monolayer; and −0.12 eV, −0.43 eV, and −0.22 eV for Ni–Nb‐doped Ti_3_C_2_O_2_ monolayer, respectively. As shown in Figure 1d, in comparison with Ti_3_C_2_O_2_ and Nb‐doped Ti_3_C_2_O_2_, the Δ*G*_H_ values for H* adsorbed at the most active site are decreased more than half after adding the surface Ni/Co atoms, suggesting an optimal HER activity after Ni/Co SAM. We also perform the Mulliken Populations Analysis (MPA), which confirms the electron transfer to O atom after Nb and Co/Ni doping (Figure S3, Supporting Information), thus enhance the HER performance.^13^

**Figure 1:**
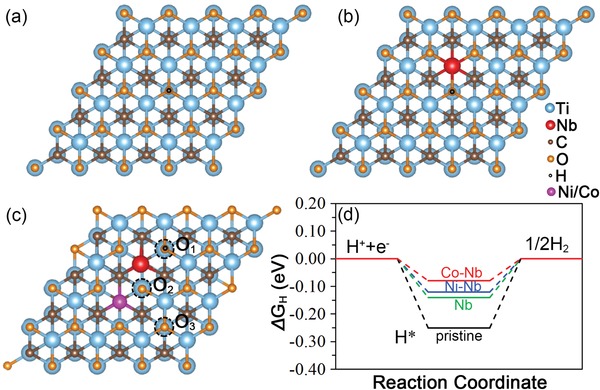
a) Atomistic configuration of pristine monolayer Ti_3_C_2_O_2_ with H* adsorption. b) Atomistic configuration of Nb doped on pristine monolayer Ti_3_C_2_O_2_ with H* adsorption. c) Atomistic configuration of Co/Ni replaced Ti atom on Nb‐doped pristine monolayer Ti_3_C_2_O_2_ and the three different H* adsorption O sites. Here the H* was adsorbed on O1 site. d) Gibbs free energies for H* adsorbed at active site show in (a–c) on M‐doped Ti_3_C_2_O_2_ monolayer.

Guided by the DFT calculations, the Nb‐doped Ti_3_C_2_T*_x_* MXene was first synthesized. The isolated Ti_2.5_Nb_0.5_C_2_T*_x_* MXene (NTM) nanosheets were obtained through a modified HF selective etching method from the bulk Ti_2.5_Nb_0.5_AlC_2_.^14^ The phase purity of each product was verified by powder X‐ray diffraction (XRD) technique first. As shown in Figure S4 (Supporting Information), the XRD pattern of as‐synthesized Ti_2.5_Nb_0.5_AlC_2_ is in accordance with the corresponding Ti_3_AlC_2_ phase (JCPDS No. 52‐0875). After delamination, most of the peaks disappear, whereas a new intensified peaks of (002) plane at 2θ ≈ 6.86° from NTM phase appears, indicating the successful exfoliation of Ti_2.5_Nb_0.5_AlC_2_.^15^ The morphology of exfoliated NTM nanosheets was then characterized by scanning electron microscopy (SEM, Figure S5a,b, Supporting Information). From the SEM images, the freeze‐dried NTM nanosheets were well exfoliated into isolated sheets after ultrasonic treatment. The homogeneous distribution of Ti, Nb, O, and F in the NTM nanosheets is confirmed by the energy dispersive X‐ray spectroscopy (EDX) elemental mapping (Figure S5c, Supporting Information). Furthermore, atomic force microscopy (AFM) was applied to get the information of the thickness of exfoliated NTM nanosheets. As shown in Figure S5d (Supporting Information), the observed Ti_2.5_Nb_0.5_C_2_T*_x_* nanosheets have an average step height of around 7.62 nm, which indicated the multilayered structures.

The morphology and phase of as‐obtained NTM nanosheets were also confirmed by the transmission electron microscopy (TEM, Figure S6, Supporting Information). As shown in Figure S6a,b (Supporting Information), the NTM nanosheets show a multilayered morphology, which is in accordance with the AFM results. The selected area electron diffraction (SAED) pattern of stacked NTM nanosheets is inserted in Figure S6a (Supporting Information), which shows dotted rings, indicating the crystalline nature and different orientation of the NTM nanosheets. The high‐resolution TEM (HRTEM) image of Ti_2.5_Nb_0.5_C_2_T*_x_* nanosheet is shown in Figure S6c (Supporting Information), which reveals a series of lattice planes. By applying the inverse fast Fourier transformation (FFT), two lattice planes with interlattice distances of 0.265 and 0.218 nm can be identified, which correspond to the {100} and {105} planes of the NTM, respectively.

The SAM was performed via a thermal reduction process based on the self‐assembled layered double hydroxides (LDHs)–MXene precursors (for detail, see Supporting Information). The obtained nanocomposites were denoted as Ni@NTM, Ni_0.9_Co_0.1_ @ NTM, Ni_0.8_Co_0.2_ @ NTM, and Ni_0.7_Co_0.3_ @ NTM in according with the mole ratio of Ni and Co in the starting materials for Ni/Co‐LDHs. The mole ratio of Ni and Co in the products was also confirmed by the EDX analysis (Table S1, Supporting Information). After SAM, the phase purity of each product was also verified by XRD. As shown in Figure S7 (Supporting Information), with the increase of Co:Ni ratio, the peaks of (111) and (200) planes from Ni/Co alloy (JCPDS No. 89‐7128) weakened and broaden, indicating the decrease of particle size. **Figure** **2** shows the SEM, TEM, and HRTEM results of the Ni_0.9_Co_0.1_ @ NTM nanohybrid. As shown in Figure 2a,b, the nanohybrid maintains a discrete nanosheet morphology, in which uniform nanodots can be observed on the NTM nanosheets. The HRTEM image and the corresponding inverse FFT image of the Ni_0.9_Co_0.1_ alloy nanoparticles are shown in Figure 2c,d. As labeled in Figure 2d, the Ni_0.9_Co_0.1_ alloy nanoparticle presents an average diameter of about 5 nm with good crystallinity. The interlattice distances of 0.203 nm can be ascribed to the {111} plane of the Ni metal phase (JCPDS No. 89‐7128). The other planes of Ni_0.9_Co_0.1_ alloy nanoparticle were labeled in the corresponding SEAD pattern (inserted in Figure 2d). The presence and homogeneously distribution of Ti, Nb, O, F, Ni, and Co elements in the nanohybrid are also confirmed by the EDX elemental mapping (Figure S8, Supporting Information).

**Figure 2:**
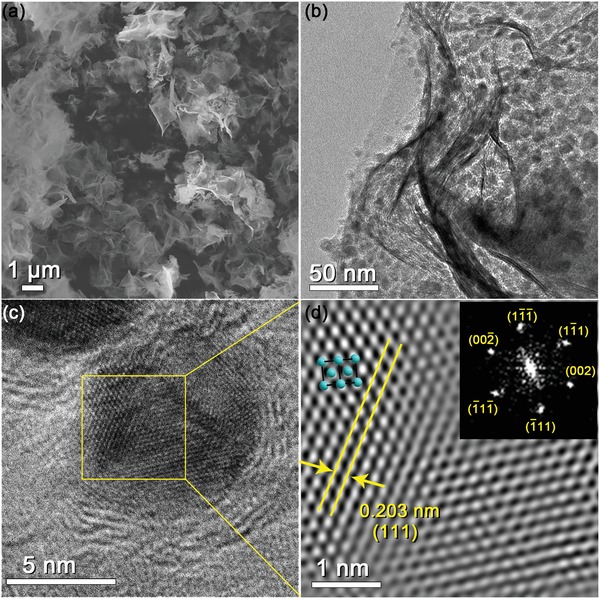
a) SEM image and b) TEM images of the Ni_0.9_Co_0.1_ @ NTM nanohybrid. c) HRTEM image of the Ni_0.9_Co_0.1_ @ NTM nanohybrid and d) the inverse FFT image of the selected area in (c).

In order to further investigate the surface chemical composition and chemical bonding of the as‐prepared NiCo@NTM nanohybrids, X‐ray photoelectron spectroscopy (XPS) of the Ni_0.9_Co_0.1_ @ NTM nanohybrid were acquired (**Figure** **3**). The high‐resolution XPS signals of Ti 2p, Nb 3d, F 1s, and O 1s spectra indicate the presence of NTM MXene after the SAM (Figure 3a–d). In the Ti 2p spectrum, six peaks centered at 464.2, 462, 459, 457.3, 456.2, and 455.3 eV can be deconvoluted. The peaks at 464.2, 462, 456.2, and 455.3 eV are related to the C—Ti—O/F and C—Ti binding.^16^, ^17^, ^18^, ^19^ The two peaks at 459 and 457.3 eV are assigned to the TiO_2−_ *_x_*F*_x_* and TiO_2_ species.^16^, ^17^, ^18^, ^20^ The Nb 3d spectrum also can be deconvoluted into six peaks, which related to the Nb–C (207 and 203.9 eV), Nb–C*_x_*O*_y_*F*_z_* (208.4 and 204.7 eV), and Nb_2_O_5_ (210.3 and 207.7) species.^19^ In the O 1s spectrum, five peaks centered at 533.7, 532.5, 531.7, 530.7, and 530.1 eV can be deconvoluted. The peaks at 531.7 and 530.7 eV can be ascribed to the surface C–Ti/Nb–OH and C–Ti/Nb–O*_x_* species, respectively.^17^, ^19^ The peak centered at 530.1 eV is related to the surface metal oxides species such as TiO_2−_ *_x_*, Nb_2_O_5_, and Ni/Co–O binding.^21^ The peaks centered at 533.7 and 532.5 eV are related to the surface adsorbed water (H_2_O_ads_) and oxygen (O_ads_) species, respectively.^17^, ^18^ The F 1s spectrum presents a peak centered at 685.3 eV, which can be assigned to the C–Ti/Nb–F*_x_* binding.^19^ To explore the surface group variation of the NTM before and after thermal reduction, XPS spectrum of the pristine NTM is acquired (Figure S9, Supporting Information, for details, see Supporting Information). As a result, the amount of surface oxygen‐containing species increases slightly after the thermal treatment, but the amounts of C–Ti/Nb–OH, C–Ti/Nb–F*_x_*, and C–Ti/Nb–F*_x_* species remain similar after the thermal treatment, indicating the surface stability of NTM under the thermal treatment condition.

**Figure 3:**
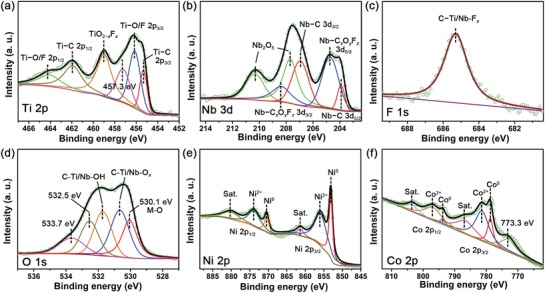
Chemical composition and bonding configuration of the as‐synthesized Ni_0.9_Co_0.1_ @ NTM nanohybrid. XPS spectra of a) Ti 2p, b) Nb 3d, c) F 1s, d) O 1s, e) Ni 2p, and f) Co 2p from the Ni_0.9_Co_0.1_ @ NTM nanohybrids.

The high‐resolution XPS spectrum is also applied to identify the chemical bonding characteristics of Ni/Co alloy. As shown in Figure 3e, the Ni 2p spectrum can be deconvoluted into six peaks. The two peaks at 853.1 and 870.5 eV are belonged to the 2p_3/2_ and 2p_1/2_ core levels of Ni^0^.^22^, ^23^ While the two peaks at 856.1 and 874 eV indicate the existence of Ni^2+^.^24^ The satellite peaks of the Ni 2p_1/2_ and 2p_3/2_ core levels are located at 880.2 and 861.4 eV, respectively.^23^ The Co 2p spectrum was shown in Figure 3f, which were deconvoluted into seven peaks located at 803.7, 796.7, 793.7, 786.9, 781.7, 778.7, and 773.3 eV. The peaks at 793.7 and 778.7 eV correspond to the Co 2p_1/2_ and Co 2p_3/2_ of Co^0^, respectively.^23^, ^25^, ^26^ While the peaks at 796.7 and 781.7 eV were related to the Co^2+^ species.^27^ The satellite peaks of the Co 2p_1/2_ and 2p_3/2_ core levels are located at 803.7 and 786.9 eV, respectively.^25^ The peak at 773.3 eV was related to the Co LMM Auger peaks.^28^

The electrocatalytic HER activities of as‐synthesized NiCo@NTM nanohybrids were evaluated by linear sweep voltammetry (LSV) in 1 m KOH solution. The ohmic potential drop was also corrected. As shown in **Figure** **4**a, the HER polarization curves of the series NiCo@NTM nanohybrids, Ni@NTM nanohybrid, NTM, and 10% Pt/C were plotted. Particularly, the Ni_0.9_Co_0.1_@NTM reveals an overpotential (η) of 43.4 mV to reach a current density of 10 mA cm^−2^, which is comparable to the commercial 10% Pt/C electrocatalyst (34.4 mV@10 mA cm^−2^) and much lower than the bare NTM material (516.4 mV@10 mA cm^−2^). Moreover, the Ni_0.9_Co_0.1_ @ NTM sample also depicts the lowest overpotential among all tested the NiCo @ NTM samples with varied Ni:Co ratio including Ni_0.8_Co_0.2_ @ NTM (η = 121.0 mV), Ni_0.7_Co_0.3_ @ NTM (η = 184.5 mV), and Ni @ NTM (η = 177.4 mV). The low overpotential of the as‐prepared Ni_0.9_Co_0.1_ @ NTM nanohybrid is not only close to the commercial 10% Pt/C catalyst, but also comparable or even better than some of the state‐of‐the‐art non‐noble metal‐based HER electrocatalysts, such as MoNi_4_/MoO_2_ @ Ni foam (η = 15 mV),^29^ V‐Co_4_N NS (η = 37 mV),^30^ Mo_2_N‐Mo_2_C/HGr3 (η = 154 mV),^31^ and (NEt_4_)_2_[Mo_3_S_7_Br_3_(*p*‐BDT)_1.5_] (η = 89 mV).^32^ It is also worth to note that the overpotential of all the NiCo@NTM nanohybrids reported in this work are much lower than the reported Ni/Co alloy growth on reduced graphene oxide (η = 512 mV in 0.5 m H_2_SO_4_) and graphene (η > 1.2 V vs SCE in 6 m KOH).^33^ The HER kinetics of the as‐prepared electrocatalysts are also probed by the corresponding Tafel plots (Figure 4b).^34^, ^35^ As shown in Figure 3b, the Tafel slopes are 116, 118, 145, 185, and 154 mV per decade for Ni_0.9_Co_0.1_ @ NTM, Ni_0.8_Co_0.2_ @ NTM, Ni_0.7_Co_0.3_ @ NTM, Ni@NTM, and bare NTM, respectively. The Tafel slope of Ni_0.9_Co_0.1_ @ NTM was coincident with the theoretical value of typical polycrystalline nickel electrode, suggests an HER kinetics of Volmer–Heyrovsky mechanism.^36^

**Figure 4:**
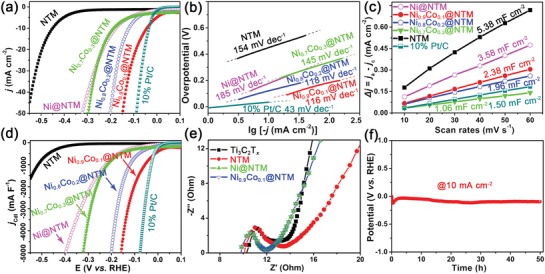
a) HER polarization curves of the series NiCo@NTM nanohybrids, Ni@NTM nanohybrid, NTM, and 10% Pt/C in 1 m KOH with a scan rate of 5 mV s^−1^. b) Corresponding Tafel plots of the series samples. c) The charging current density differences plotted against scan rates of the as‐prepared series NiCo@NTM and Ni@NTM nanohybrids. The linear slope is equivalent to twice of electrochemical double‐layer capacitance (*C*_dl_). d) HER polarization curves shown in (a) normalized by the *C*_dl_. e) Nyquist plots of the electrodes modified by Ti_3_C_2_T*_x_*, NTM, Ni@NTM, and Ni_0.9_Co_0.1_@NTM measured at zero overpotential versus RHE. f) The chronopotentiometric curves of the Ni_0.9_Co_0.1_@NTM nanohybrid under static current density (10 mA cm^−2^) over 50 h.

Since usually there is a positive correlation between the catalytic activity and the active surface area,^26^, ^35^, ^37^ the electrochemical doublelayer capacitance (*C*_dl_) of the as‐prepared NiCo @ NTM nanohybrids were determined to estimate their electrochemically effective surface area (ECSA).^38^ As shown in Figure 4c and S10 (Supporting Information), the NTM has the largest *C*_dl_ of 5.38 mF cm^−2^. After SAM, the *C*_dl_ of Ni @ NTM and three NiCo@NTM compounds were decreased along with the increase of Co content (3.58, 2.38, 1.96, and 1.06 mF cm^−2^ for Ni@NTM, Ni_0.9_Co_0.1_ @ NTM, Ni_0.8_Co_0.2_ @ NTM, and Ni_0.7_Co_0.3_ @ NTM, respectively). This might be ascribed to the decrease of the lateral size of Ni/Co‐LDHs precursors when increasing the Co content (Figure S11, Supporting Information), which makes the NTM nanosheets easier to restack. The current density *j* of 10% Pt/C, NTM, Ni@NTM, and the three NiCo@NTM compounds were then normalized by the *C*_dl_.^39^ Since for Pt/C the overpotential at $j_{C_{\mathrm{dl}}}$ = 1000 mA F^−1^ (28.7 mV) is approximate to *j* = 10 mA cm^−2^, the overpotential for each sample to reach the $j_{C_{\mathrm{dl}}}$ of 1000 mA F^−1^ was selected to better compare the HER activity. As a result, the Ni_0.9_Co_0.1_ @ NTM still presents the lowest overpotential of 55.5 mV to reach the $j_{C_{\mathrm{dl}}}$ of 1000 mA F^−1^ among all the prepared compounds (Figure 4d), while the bare NTM required an overpotential of 515.9 mV. Therefore, it is reasonable to suppose that the boost of HER performance after SAM was originated from the higher intrinsic activity of this 0D–2D hybrid structure. In order to further verify the effectiveness of Nb doping, the normalized LSV curves of undoped Ti_3_C_2_T*_x_* and NiCo@Ti_3_C_2_T*_x_* (denoted as NiCo @ TM) were also acquired. As shown in Figure S12a (Supporting Information), the non‐Nb‐doped samples all shows higher overpotential than the Nb‐doped one, indicates the beneficial effect of Nb doping. Also, the effect of thermal treatment on bare NTM was evaluated, which can cause the deterioration of the HER activity (Figure S12b, Supporting Information).

To further verify the charge transfer kinetics of these nanohybrids, electrochemical impedance spectra (EIS) of the as‐prepared Ti_3_C_2_T*_x_* MXene, bare NTM, Ni@NTM, and Ni_0.9_Co_0.1_ @ NTM were tested. As shown in Figure 4e, after doping and SAM, the charge transfer resistance (*R*_ct_) of the tested samples were slightly decrease: from 13.2 Ω for Ti_3_C_2_T*_x_* MXene to 12.5 Ω for bare NTM, and 11.8 Ω for both Ni @ NTM and Ni_0.9_Co_0.1_ @ NTM.[qv: 24a,29]

The chronopotentiometry curve of the Ni_0.9_Co_0.1_@NTM nanohybrid was recorded at a constant current density of 10 mA cm^−2^ over 50 h (Figure 4f). After 50 h HER process, the current density remains almost the same. The LSV curves before and after 50 h HER test are overlapped well (Figure S13, Supporting Information), which further demonstrates the excellent HER stability of the Ni_0.9_Co_0.1_ @ NTM nanohybrid. The TEM and HRTEM images of the Ni_0.9_Co_0.1_ @ NTM nanohybrid after 50 h HER test are shown in Figure S14 (Supporting Information). As shown, the unique structure of Ni_0.9_Co_0.1_ @ NTM could well remain. In addition, the interlattice distances of 0.203 nm observed under HRTEM can still match well with the {111} plane of Ni metal phase (JCPDS No. 89‐7128), indicating the good stability of the Ni_0.9_Co_0.1_ @ NTM nanohybrid (Figure S14b,c, Supporting Information). To verify that all the current is due to water decomposition, the experimental yield of H_2_ from Ni_0.9_Co_0.1_@NTM electrode in 1 h at a constant current of −10 mA were determined by gas chromatography, which showing nearly 100% Faradaic efficiency of electrolysis when compared with the theoretical plots (Figure S15, Supporting Information).

In summary, a novel Ti_3_C_2_T*_x_* MXene‐based nanohybrid with simultaneously Nb doping and surface Ni/Co alloy modification was successfully synthesized under the guidance of theoretical calculation. With the Nb doping and surface Ni/Co alloy modification, the NiCo@NTM shows an extraordinary HER activity in alkaline solution. Partially, the Ni_0.9_Co_0.1_@NTM nanohybrids presents the best HER activity in alkaline solution among all the prepared NiCo@NTM samples, which only needs an overpotential (η) of 43.4 mV to reach the current density of 10 mA cm^−2^ in 1 m KOH solution and shows good stability. The performance of the Ni_0.9_Co_0.1_ @ NTM nanohybrids is not only close to the commercial 10% Pt/C electrode, but also comparable or even better than most of the state‐of‐the‐art Pt‐free HER catalysts. In view of the facile synthesis process and chemical versatility of both MXene and transition metal alloys, the MXene‐based nanohybrids reported here are a promising non‐noble metal electrocatalyst for water–alkali electrolysis.

## Conflict of Interest

The authors declare no conflict of interest.

## Supporting information

Supplementary
