# Supplementary material for: Synergy of Nb Doping and Surface Alloy Enhanced on Water–Alkali Electrocatalytic Hydrogen Generation Performance in Ti‐Based MXene
Source: Adv Sci (Weinh). 2019 Apr 5;6(11):1900116. doi: 10.1002/advs.201900116 (PMC6548967; doi:10.1002/advs.201900116)
Supplement: Supplementary file 1 — Supplementary [file ADVS-6-1900116-s001.pdf]

Copyright WILEY-VCH Verlag GmbH & Co. KGaA, 69469 Weinheim, Germany, 2019.

Supporting Information

for *Adv. Sci.,* DOI: 10.1002/advs.201900116

Synergy of Nb Doping and Surface Alloy Enhanced on Water–Alkali Electrocatalytic Hydrogen Generation Performance in Ti-Based MXene

Cheng-Feng Du, Xiaoli Sun, Hong Yu, Qinghua Liang, Khang Ngoc Dinh, Yun Zheng, Yubo Luo, Zhiguo Wang, and Qingyu Yan**

Copyright WILEY-VCH Verlag GmbH & Co. KGaA, 69469 Weinheim, Germany, 2019.

Supporting Information

Synergy of Nb-doping and surface alloy enhanced on water-alkali electrocatalytic hydrogen generation performance in Ti-based MXene

Cheng-Feng Du, Xiaoli Sun, Hong Yu, Qinghua Liang, Khang Ngoc Dinh, Yun Zheng, Yubo Luo, Zhiguo Wang, and Qingyu Yan**

Chemicals and Characterization:

Titanium powder (99.5%), niobium powder (99.8%), aluminum powder (99%) and graphite powder (99.9%) were purchased from Alfa Aesar. Lithium fluoride (≥99.0%), ethylene glycol (anhydrous, 99.8%), Urea (99%), $Ni(NO_3)_2{\cdot}6H_2O$ (≥98.5%), $Co(NO_3)_2{\cdot}6H_2O$ (≥98%), KOH (99.99%) and Nafion solution (5 wt% in deionized water) were purchased from Sigma-Aldrich, 2-propanol (99+%) and hydrochloric acid (technical grade) was purchased from Fisher. All the chemicals were used without further purification. The high purity deionized water was purified using the Milli-Q system (Millipore, Billerica, MA, USA).

Powder X-ray diffraction (XRD) patterns were recorded on a Bruker D8 Advance powder diffractometer by using Cu $K\alpha$ radiation (λ = 0.15404 nm). Scanning electron microscopy (SEM) and energy dispersive X-ray spectroscopy (EDX) elemental mapping images were acquired on a JEOL JEM-7600F microscope. TEM images were acquired on a JEOL 2010-UHR microscope at 200 kV, XPS spectra were acquired on Kratos AXIS Supra.

Simulation details and methods

All the calculations were performed by using density functional theory (DFT) as implemented in the Vienna *ab initio* package (VASP).[1] The projector augmented wave (PAW) method was used to describe electron-ion interaction,[2] while the generalized

gradient approximation using the Perdew-Burke-Ernzerhof (PBE) functional was used to describe the electron exchange-correlation. A plane wave basis was set up to an energy cutoff of 520 eV. A 4×4 supercell of $Ti_3C_2T_x$ monolayer was used to investigate the adsorption of hydrogen. A 30 Å vacuum space were constructed to avoid the periodical image interactions between periodical interactions. The Brillouin zone was integrated using the Monkhorst-Pack scheme with 5×5×1 *k*-grid.[3] All the atomic positions and cell parameters were relaxed using a conjugate gradient minimization until the force on each atom is less than 0.01 eV/Å.

Gibbs free-energy of the adsorption atomic hydrogen was calculated using equation (1):

$$\Delta G_{\mathrm{H}} = \Delta E_{\mathrm{H}} + \Delta E_{\mathrm{ZPE}} - T\Delta S_{\mathrm{H}} \quad (1)$$

Where ΔE_{ZPE} and ΔS_{H} are the zero-point energy and entropy difference of hydrogen in the adsorbed state and the gas phase, respectively. The hydrogen adsorption energy ΔE_{H} is calculated with the following expression:

$$\Delta E_{\mathrm{H}} = E_{M+H} - E_M - \frac{1}{2}E_{\mathrm{H_2}} \quad (2)$$

where E_{M+H} and E_M are the total energy of M-doped the $Ti_3C_2F_2$ monolayer with and without H atoms adsorption, respectively. $E_{\mathrm{H_2}}$ is the energy of a gas phase hydrogen molecule.

The calculated frequencies of H_2 gas is 4345 cm^{-1}. The contribution from the configurational entropy in the adsorbed state is small and neglected. So the entropy of hydrogen adsorption as $\Delta S_H = \frac{1}{2}S_{H2}$ where S_{H_2} is the entropy of molecule hydrogen in the gas phase at standard conditions.[4] With these values the Gibbs free energy of equation (1) can be rewritten as:

$$\Delta G_{\mathrm{H}} = \Delta E_{\mathrm{H}} + 0.29 \quad (3)$$

Synthesis of and the exfoliation of $Ti_3C_2T_x$ and $Ti_{2.5}Nb_{0.5}C_2T_x$ MXene:

Ti_3AlC_2 and $Ti_{2.5}Nb_{0.5}AlC_2$ was synthesized following the approach reported by Sun et al.[5] Take $Ti_{2.5}Nb_{0.5}AlC_2$ as an example: the powder of Ti, Nb, Al and C were mix with the molar ratio of Ti:Nb:Al:C = 2.5:0.5:1.2:2, followed by ball milling for 12 h under Ar atmosphere in a 8000M Mixer/Mill® High-Energy Ball Mill. The mixture was then sintering under flowing argon (Ar) in a tube furnace for 2 h at 1450 ºC. The resulting lightly sintered brick was ground with an agate mortar and sieved through a 400 mesh sieve.

$Ti_3C_2T_x$ and $Ti_{2.5}Nb_{0.5}C_2T_x$ MXene was prepared according to the literature.[6, 7] Take $Ti_{2.5}Nb_{0.5}AlC_2$ as an example: concentrated HCl was mixed with deionized water (DI water) to prepare a 6 M solution (30 mL total). LiF (1.98 g) was added to this solution. The mixture was stirred for 5 min with a magnetic Teflon stir bar to dissolve the salt. $Ti_{2.5}Nb_{0.5}AlC_2$ powder (1 g) was carefully added over the course of 10 min to avoid initial overheating of the solution as a result of the exothermic nature of the reactions. The reaction mixture was then held at 60 ºC for 2 days, after which the mixture was washed by DI water addition, centrifugation (3500 rpm × 5 min for each cycle), and decanting, until the supernatant reached a pH of approximately 6. The precipitates were then ultra-sonicated in an ice-bath for 8 h. Finally, the supernatant were collected by centrifugation and freeze dried.

Synthesis of the Ni/Co-LDHs:

In a typical synthesis process, a mixture consists of DI water (5 mL), ethylene glycol (EG, 35 mL), and the corresponding metal nitrate and urea in a molar ratio of 1:4 were added and vigorous stirring for 1 h. The final solution was solvothermally treat at 100 ºC for 24 hours. After which the mixture was several times until the supernatant become clear and then ultra-sonicated in ethanol for 1 h follow by washed and centrifuged in DI water again. The precipitates were then re-dispersed in DI water for further used.

Synthesis of the $Ni_{1-x}Co_x$ alloy-decorated Ti-MXene (NiCo@TM) and Nb-doped Ti-MXene (NiCo@NTM):

The $Ni_{1-x}Co_x$ alloy-decorated Ti-MXene (NiCo@TM) and Nb-doped Ti-MXene (NiCo@NTM) were synthesized through a self-assemble follow by low temperature reduction process. Briefly, 40 mg of the MXene was dispersed in DI water to form an aqueous solution, then the solution was dropwise added into Ni/Co-LDHs solution which contains 1 mmol Ni/Co-LDHs under vigorous stirring and Ar bubbling, then the mixture was ultra-sonicated for 1h. After the self-assemble process, the precipitates were collected by centrifugation and freeze dried. The obtained nanocomposites were annealed in 5% H_2/Ar atmosphere at 400 ºC for 30 min with a heating rate of 3 ºC min^{-1}.

Electrode preparation and electrochemical testing:

Active materials (2.0 mg, e.g. $Ti_3C_2T_x$ MXene, NiCo@TM, $Ti_{2.5}Nb_{0.5}C_2T_x$ MXene, NiCo@NTM nanocomposites or 10% Pt/C) was mixed with acetylene black (0.5 mg) and Nafion (5 wt %, 25 µL) aqueous solution, DI water (25 µL) and 2-propanol (950 µL), followed by ultrasonication for 60 min. The catalyst dispersion (30 µL) was deposited onto the surface of a glassy carbon rotating disk electrode (GC-RDE, with a diameter of 5 mm) and dried under ambient condition. The mass loading was calculated to be around 0.38 mg cm^{-2}. Cyclic voltammetry (CV), electrochemical impedance spectroscopy (EIS) and linear sweep voltammetry (LSV) measurements were conducted in a three-electrode system on an electrochemical station (Solartron) with an electrode rotation speed of 2000 rpm. A graphite rod was used as the counter electrode, a 1 m Hg/HgO electrode was served as the reference electrode, and the catalyst-loaded GC electrode was used as the working electrode. All measured potentials *vs*. Hg/HgO were converted to a reversible hydrogen electrode (RHE) scale based on the Nernst equation below:

$$V_{RHE}\ (V) = V_{app}\ (V) + 0.0592\times pH + V^{\theta}_{Hg/HgO}\ (V) \quad (4)$$

where V_{RHE} is the applied potential *vs.* RHE; V_{app} is the applied potential *vs.* reference electrode, pH is the pH of the electrolyte, and $V^{\theta}_{Hg/HgO}$ is the standard potential of the Hg/HgO reference electrode.

For HER testing, the LSV measurements were performed at a scan rate of 5 mV s^{-1}. All LSV curves were corrected with 90% *iR*-compensation. By plotting overpotential η *vs.* logarithm of current density from polarization curves, Tafel slopes are obtained.

EIS measurements were conducted also in 1 m KOH at an open-circuit potential *vs*. RHE by applying an AC voltage with an amplitude of 5 mV in a frequency range of 0.01 Hz to 100 kHz. The electrochemically effective surface area (ECSA) was estimated from the electrochemical double-layer capacitance (C_{dl}) of the films. The C_{dl} was determined by a simple CV method. The CV was conducted in a potential window (0.1 to 0.3 V *vs*. RHE) at various scan rates of 10, 20, 30, 40, 50, and 60 mV s^{-1}. Then capacitive current $\Delta j = j_a - j_c$ at 0.2 V *vs.* RHE was plotted against various scan rates, while the slope obtained was divided by two to acquire the C_{dl} value.

Faradaic efficiency of the $Ni_{0.9}Co_{0.1}$@MXene electrode was done in an Ar-purged close H-shape cell (counter electrode was put in different chamber). The headspace of the chamber containing working electrode is 20 mL. A constant current of −10 mA was applied for 60 min. The H_2 gas generated was determined by taking 500 μL of the gas sample in the chamber containing working electrode to gas chromatography. The Faradaic efficiency was then calculated as the ratio of measured amount of H_2 and theoretical amount of H_2 (based on Faraday's law).

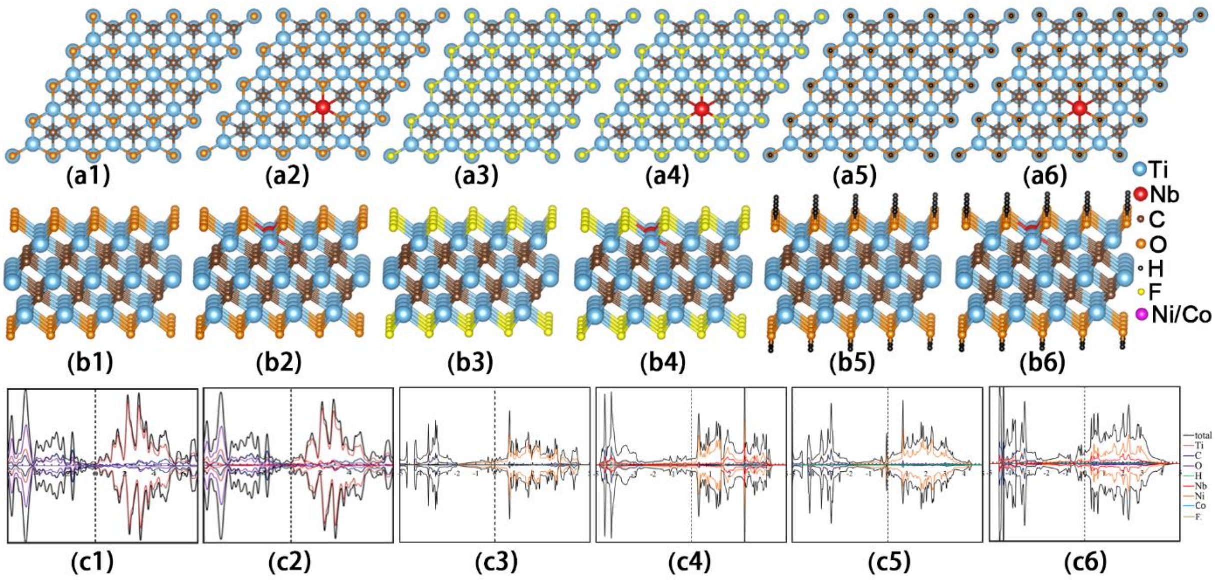

Figure S1. (a1/b1-a6/b6) Top/Side view of $Ti_3C_2O_2$, Nb-doped $Ti_3C_2O_2$, $Ti_3C_2F_2$, Nb-doped $Ti_3C_2F_2$, $Ti_3C_2(OH)_2$, and Nb-doped $Ti_3C_2(OH)_2$ monolayer. (c1~c6) Total DOS and PDOS of $Ti_3C_2O_2$, Nb-doped $Ti_3C_2O_2$, $Ti_3C_2F_2$, Nb-doped $Ti_3C_2F_2$, $Ti_3C_2(OH)_2$, and Nb-doped $Ti_3C_2(OH)_2$ monolayer. The Fermi energy is set zero.

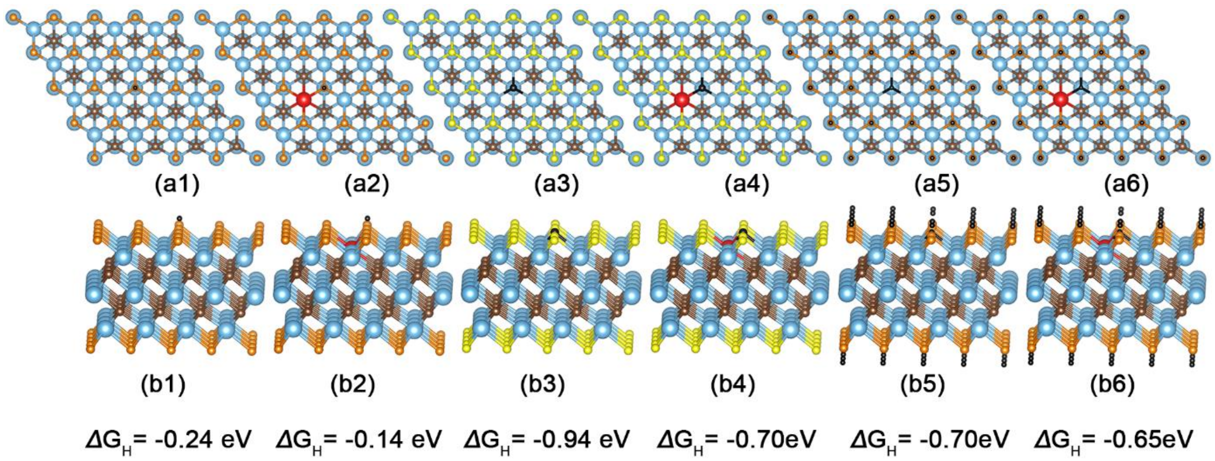

Figure S2. (a1/b1-a6/b6) Top/Side view of $Ti_3C_2O_2$, Nb-doped $Ti_3C_2O_2$, $Ti_3C_2F_2$, Nb-doped $Ti_3C_2F_2$, $Ti_3C_2(OH)_2$, and Nb-doped $Ti_3C_2(OH)_2$ monolayer with H* adsorption.

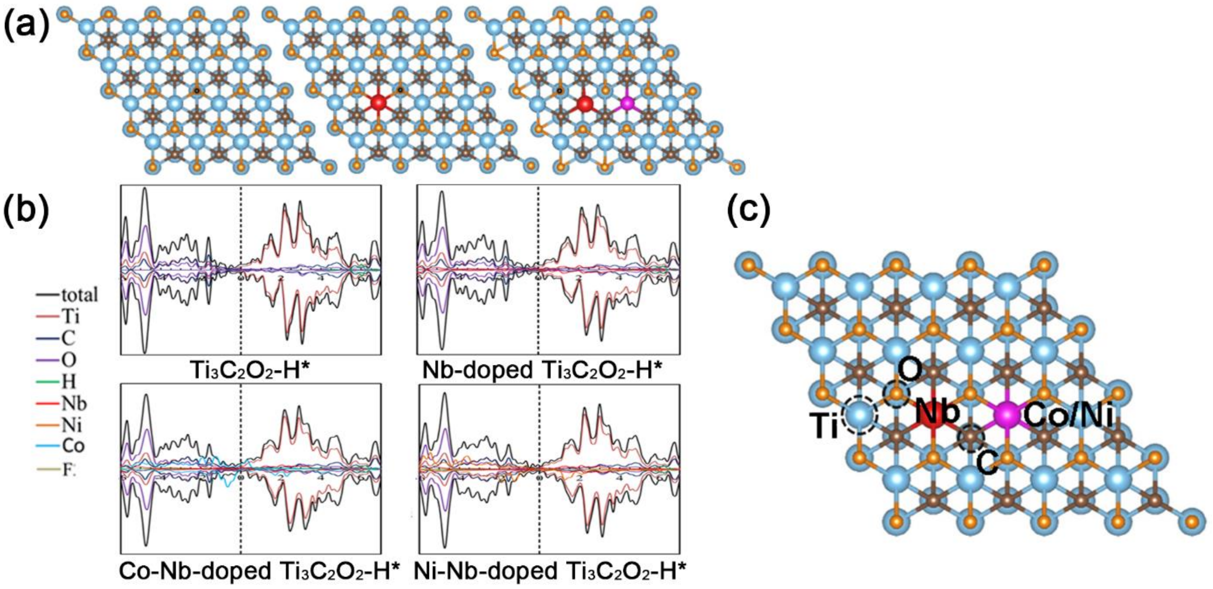

Figure S3. (a) Top view of pristine $Ti_3C_2O_2$, Nb-doped $Ti_3C_2O_2$ and Co/Ni-Nb-doped $Ti_3C_2O_2$ monolayer with H* adsorption; (b) DOS and PDOS of pristine $Ti_3C_2O_2$, Nb-doped $Ti_3C_2O_2$, Co-Nb-doped $Ti_3C_2O_2$ and Ni-Nb-doped $Ti_3C_2O_2$ monolayer with H* adsorption. The Fermi energy is set zero. (c) The electron transfer between different atoms nearby doped atoms in the Co/Ni-Nb-doped $Ti_3C_2O_2$ monolayer.

As shown in Figure S3a-b, from the DOS and PDOS of pristine $Ti_3C_2O_2$, Nb-doped $Ti_3C_2O_2$, Co-Nb-doped $Ti_3C_2O_2$ and Ni-Nb-doped $Ti_3C_2O_2$ monolayer with H* adsorption, the Nb 4d state was suggested as an electron donor. After Co/Ni-doping, the Nb 4d state donate more electron. However, the doped Co/Ni 3d states nearby Fermi energy level are not the electron donor. That endows the O atom as an electron acceptor become more active, and explain why the O_1 site is more active than O_2 site.

The result is also consistent with the Mulliken Populations Analyse. As shown in Figure S3c, O will get additional 0.014|e|, 0.017|e| and 0.017|e| after Nb-, Co-Nb- and Ni-Nb-doped $Ti_3C_2O_2$ monolayer than that of pristine $Ti_3C_2O_2$ monolayer. Ti will lose more 0.019|e|, 0.012|e| and 0.014|e| after Nb-, Co-Nb- and Ni-Nb- doping. Nb will lose more 0.041|e| and 0.043|e| after Co and Ni doping. C will get additional 0.003|e|, 0.015|e| and 0.042|e| after Nb-, Co-Nb- and Ni-Nb- doping. And after H* adsorption, C will lose 0.008|e|, 0.021|e|, 0.051|e|, 0.029|e| for pristine, Nb-doped, Co-Nb-doped and Ni-Nb-doped $Ti_3C_2O_2$ monolayer, respectively. The Co/Ni doping enhance the electron transfer kinetics and improves the HER performance.

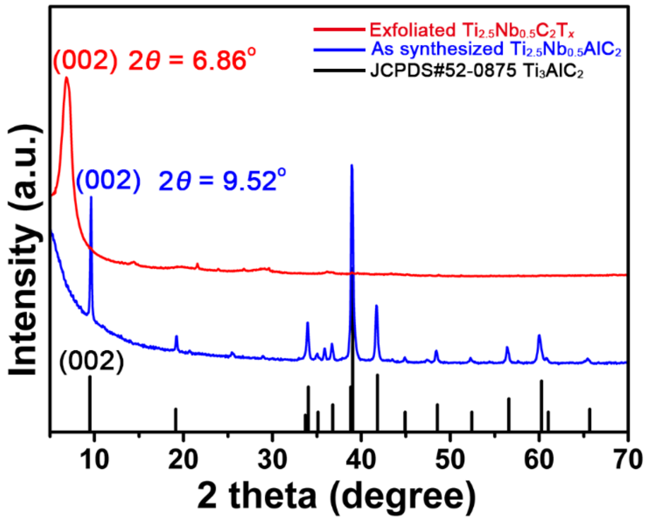

Figure S4. XRD patterns of $Ti_{2.5}Nb_{0.5}AlC_2$ powder and the isolated $Ti_{2.5}Nb_{0.5}C_2T_x$ nanosheets.

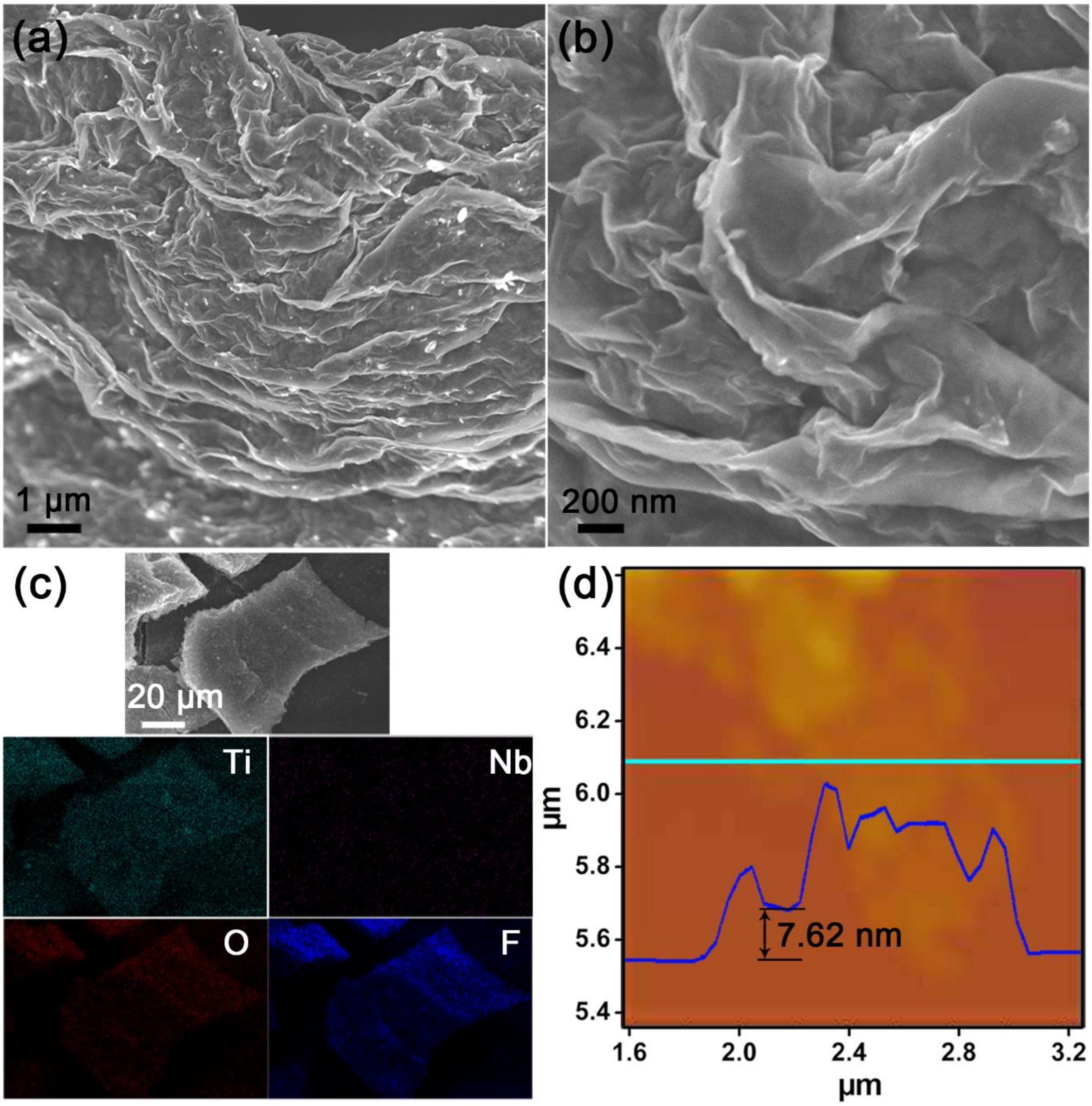

Figure S5. (a) The low-magnification SEM image and (b) high-magnification SEM images of the freeze-dried $Ti_{2.5}Nb_{0.5}C_2T_x$ nanosheets. (c) The EDX elemental mapping of the freeze-dried $Ti_{2.5}Nb_{0.5}C_2T_x$ nanosheets. (d) AFM images of the exfoliated $Ti_{2.5}Nb_{0.5}C_2T_x$ nanosheets.

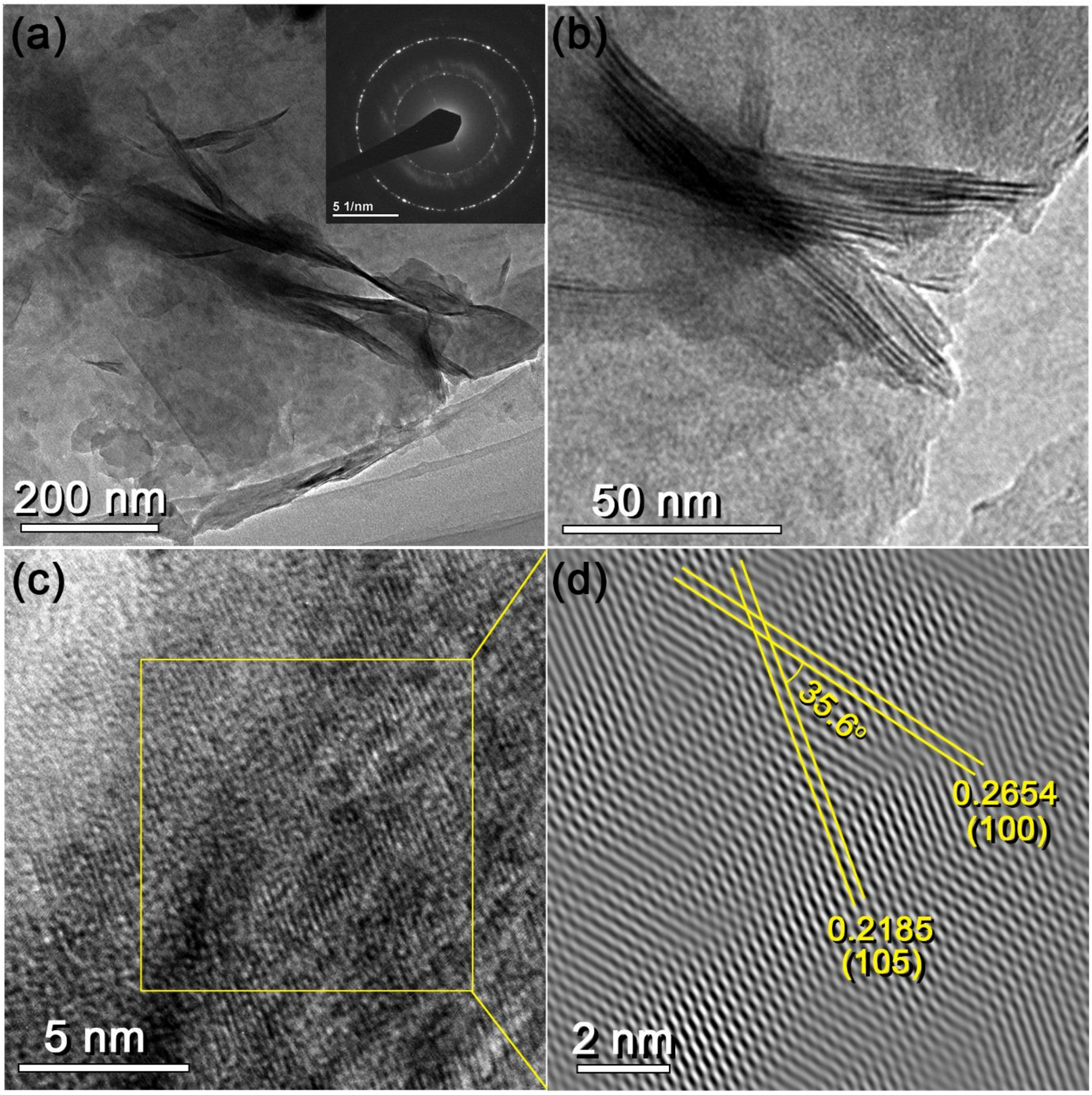

Figure S6. (a) Low-magnification TEM image of the freeze dried $Ti_{2.5}Nb_{0.5}C_2T_x$ nanosheets. Inserted shows the SAED pattern. (b) Side-view TEM image on the edge and (c) HRTEM images of the freeze dried $Ti_{2.5}Nb_{0.5}C_2T_x$ nanosheets. (d) The inverse FFT image of the selected area in (c).

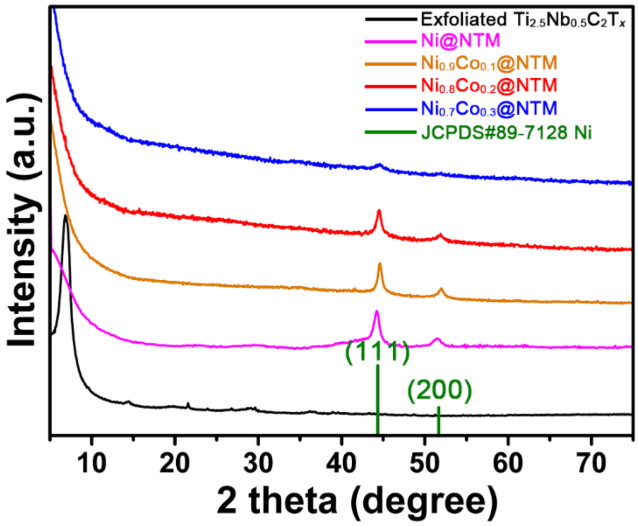

Figure S7. XRD patterns of the NiCo@NTM nanoshybrids with different Co additions.

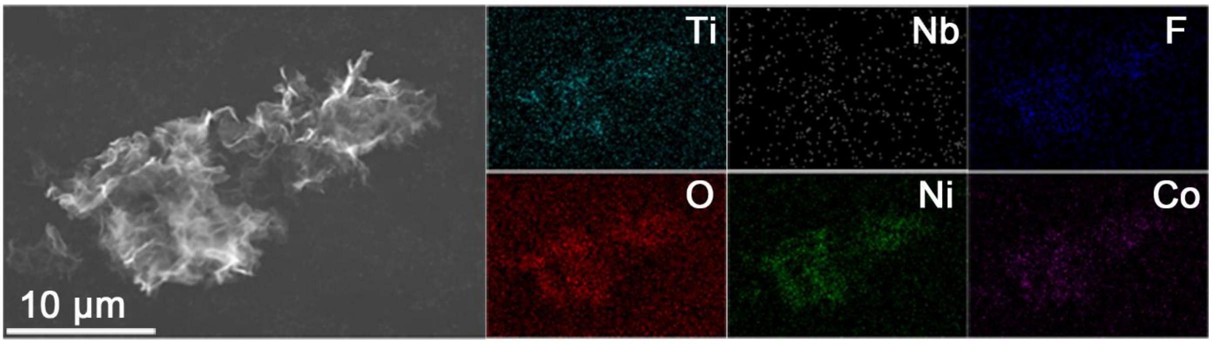

Figure S8. The EDX elemental mapping of $Ni_{0.9}Co_{0.1}$@NTM.

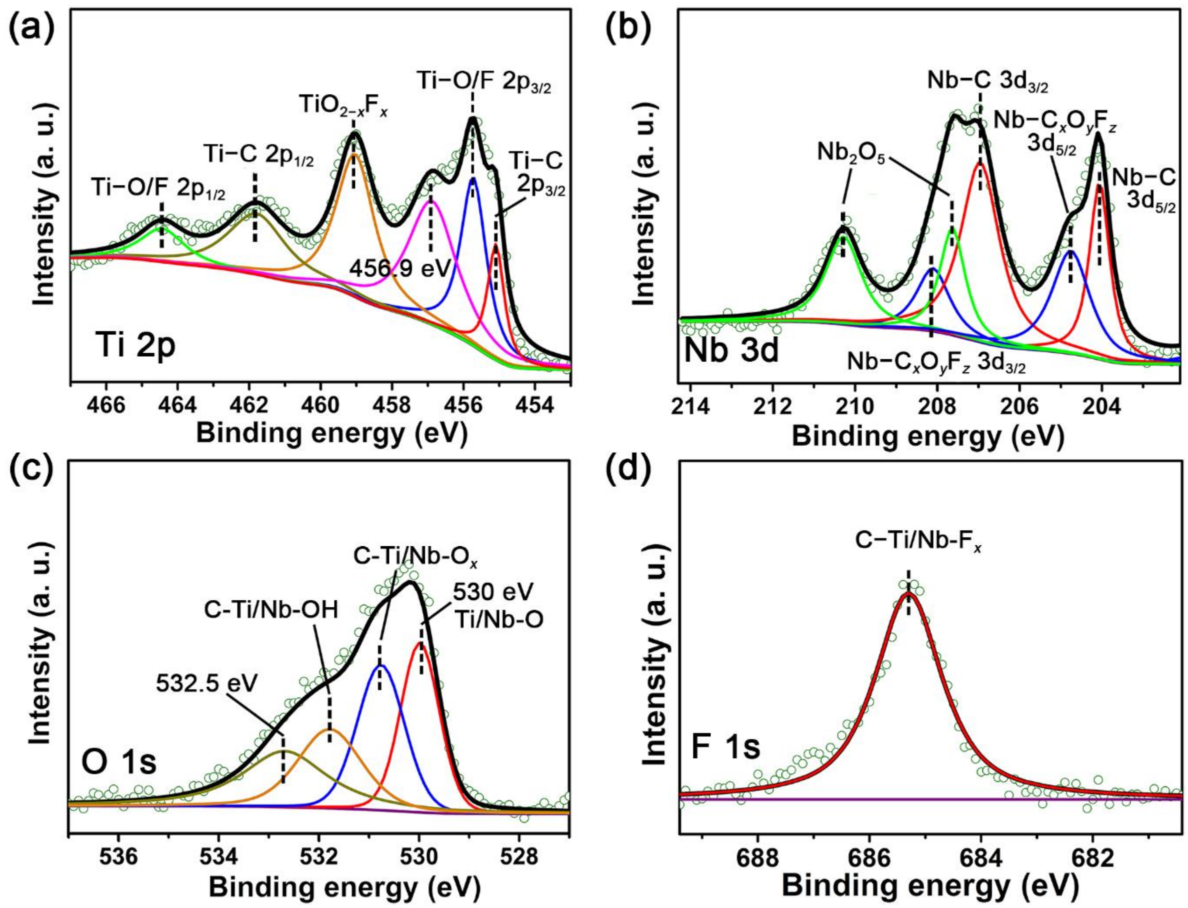

Figure S9. Chemical composition and bonding configuration of the pristine $Ti_{2.5}Nb_{0.5}C_2T_x$ nanosheets. XPS spectra of (a) Ti 2p, (b) Nb 3d, (c) O 1s and (d) F 1s from the pristine $Ti_{2.5}Nb_{0.5}C_2T_x$ nanosheets.

For the pristine $Ti_{2.5}Nb_{0.5}C_2T_x$ nanosheets, the Ti 2p spectrum can be deconvoluted into six peaks centered at 464.5, 461.8, 459, 456.9, 455.7 and 455.1 eV. The peaks at 464.5, 461.8, 455.7 and 455.1 eV are related to the C–Ti–O/F and C–Ti binding.[8-11] The two peaks at 459 and 456.9 eV are assigned to the $TiO_{2-x}F_x$ and TiO_2 species.[8-10, 12] The Nb 3d spectrum also can be deconvoluted into six peaks, which related to the Nb–C (207 and 204.1 eV), Nb–$C_xO_yF_z$ (208.1 and 204.8 eV), and Nb_2O_5 (210.3 and 207.6) species. In the O 1s spectrum, four peaks centered at 532.7, 531.8, 530.8 and 530 eV can be deconvoluted. The peaks at 531.8 and 530.8 eV can be ascribed to the surface C–Ti/Nb–OH and C–Ti/Nb–O_x species, respectively.[9, 11] The peak centered at 530 eV is related to the surface metal oxides species such as TiO_{2-x} and Nb_2O_5.[13, 14] The peaks centered at 532.5 eV are related to the surface adsorbed oxygen (O_{ads}) species. The F 1s spectrum presents a peak centered at 685.3 eV, which in accordance with the C–Ti/Nb–F_x binding after thermal treatment.[11]

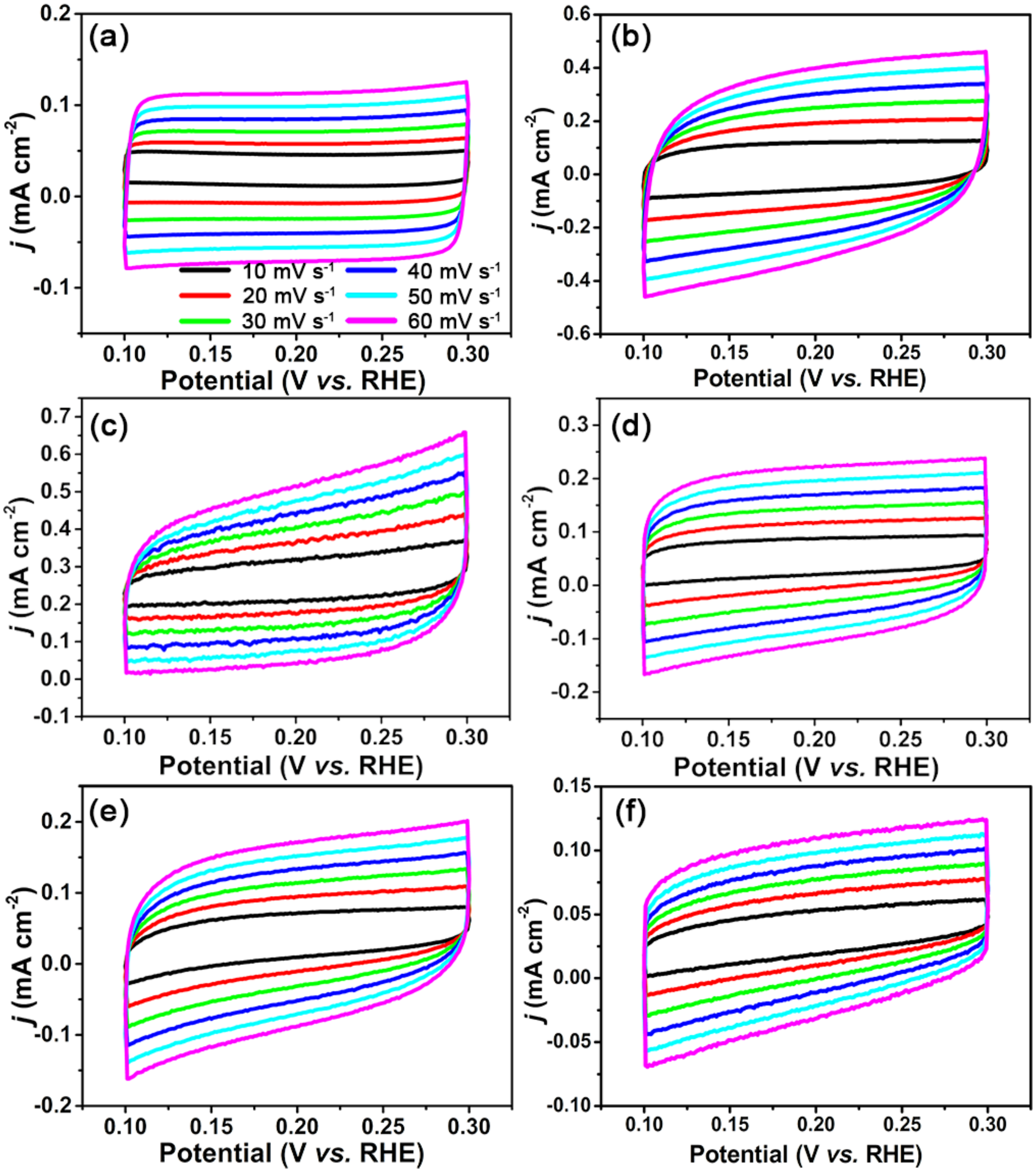

Figure S10. Cyclic voltammograms (CV) of (a) 10% Pt/C, (b) $Ti_{2.5}Nb_{0.5}C_2T_x$ MXene, (c) Ni@NTM, (d) $Ni_{0.9}Co_{0.1}$@NTM, (e) $Ni_{0.8}Co_{0.2}$@NTM, and (f) $Ni_{0.7}Co_{0.3}$@NTM are taken in a potential window (0.1 to 0.3 V *vs.* RHE) at various scan rates of 10, 20, 30, 40, 50, and 60 mV s^{-1} in 1.0 M KOH solution.

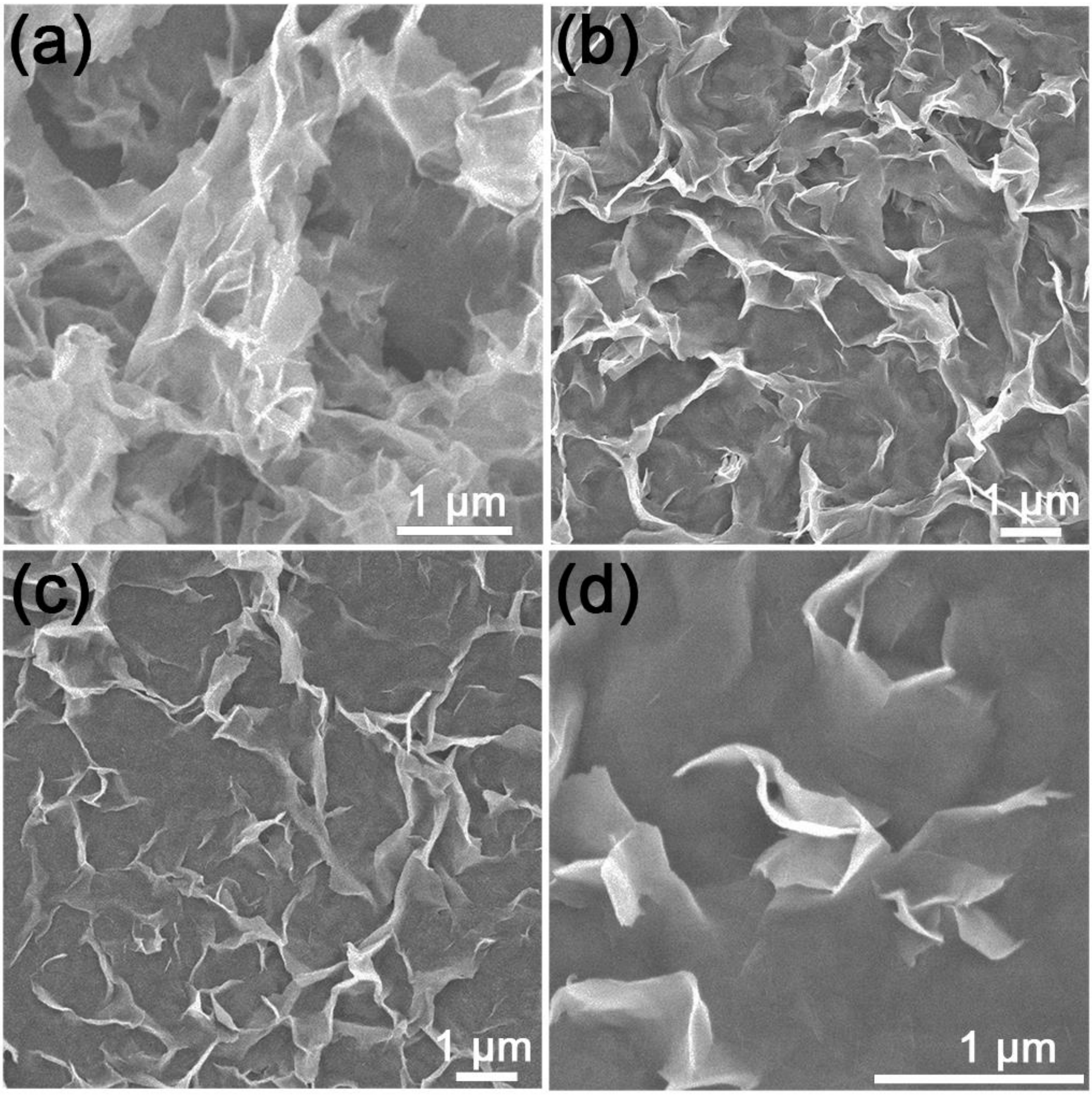

Figure S11. SEM images of the Ni/Co-LDHs with different Co additions: (a) pure $Ni(OH)_2$, (b) 10% Co, (c) 20% Co, and (d) 30% Co in mole ratio.

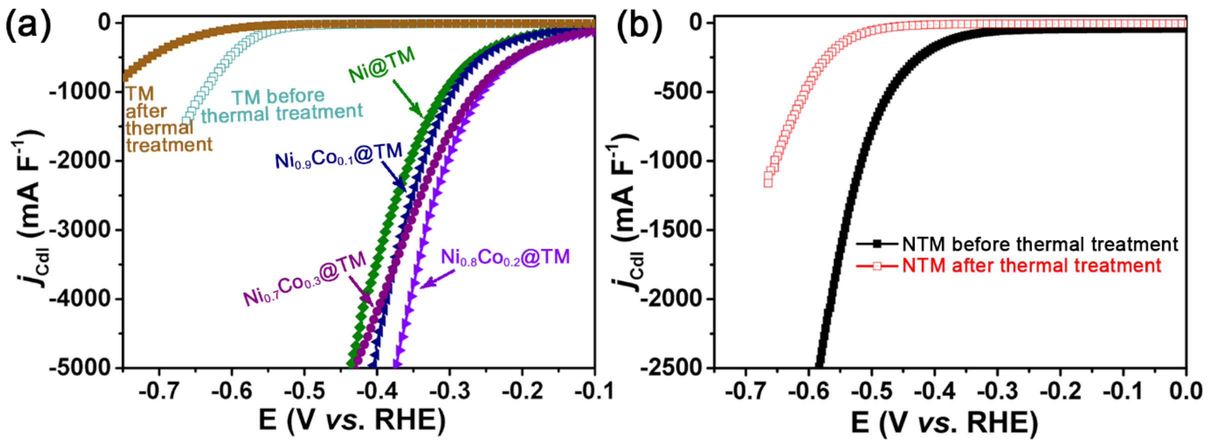

Figure S12. (a) Normalized LSV curves of the bare $Ti_3C_2T_x$ MXne and the series NiCo@TM nanohybrids in 1 m KOH with a scan rate of 5 mV s^{-1}. (b) Normalized LSV curves of the bare NTM before and after the thermal treatment in 1 m KOH with a scan rate of 5 mV s^{-1}.

As shown in Figure S12a, for all the non-Nb-doped samples, the overpotential was 307.5, 295.5, 255.5, 263.3 and 683.4 mV for the Ni@TM, $Ni_{0.9}Co_{0.1}$@TM, $Ni_{0.8}Co_{0.2}$@TM, $Ni_{0.7}Co_{0.3}$@TM and pristine TM to reach the j_{Cdl} of 1000 mA F^{-1}, respectively. While after thermal treatment, the overpotential of pristine TM was further increased to about 767.5 mV. The same phenomenon can also be observed on NTM species (Figure S12b), after thermal treatment, the overpotential of pristine NTM to reach the j_{Cdl} of 1000 mA F^{-1} was decreased from 515.9 to 650.2 mV.

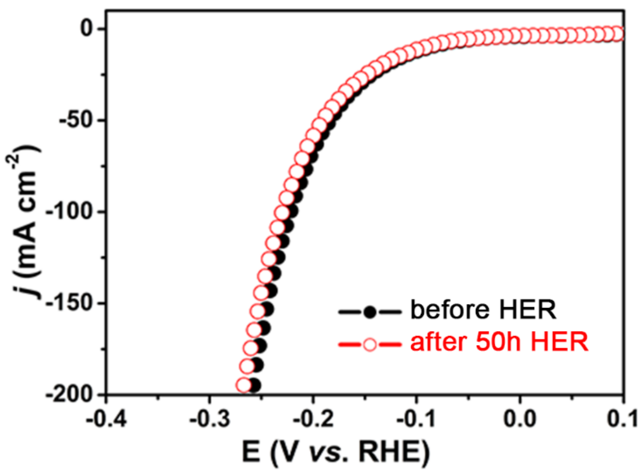

Figure S13. The LSV curves for the $Ni_{0.9}Co_{0.1}$@NTM nanohybrid before and after the 50 hours' time-dependent testing.

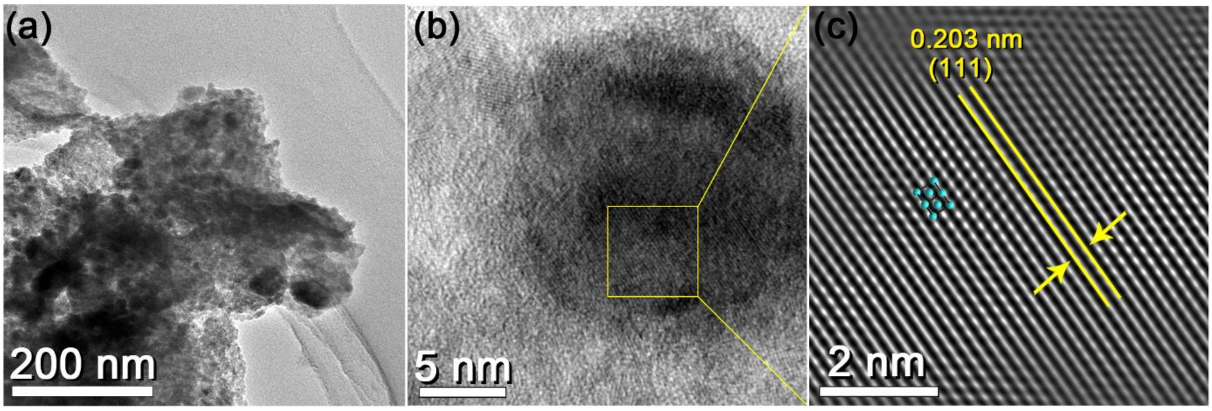

Figure S14. (a) TEM and (b) The HRTEM images of the $Ni_{0.9}Co_{0.1}$@NTM nanohybrid after HER for 50 h. (c) The inverse FFT image of the selected area in (b). The crystalline $Ni_{0.9}Co_{0.1}$ nanoparticle with a well-defined grain boundary is reserved, indicated the good stability.

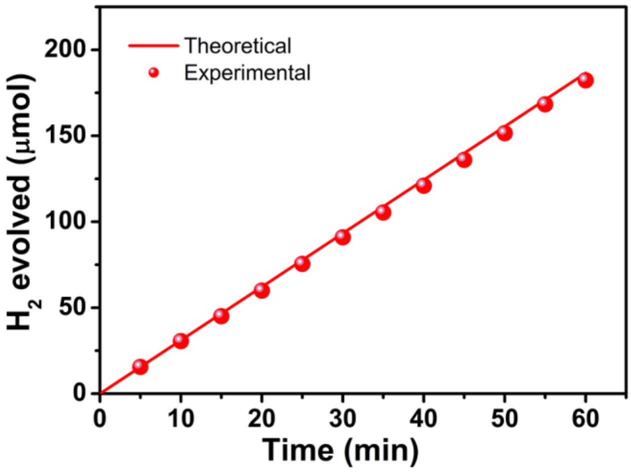

Figure S15. The Faradaic efficiency of (a) H_2 generation over $Ni_{0.9}Co_{0.1}$@NTM electrode at a current of −10 mA.

Table S1. Average contents of Ni and Co in each NiCo@NTM sample (Atomic%).

Atomic%	$Ni_{0.9}Co_{0.1}$@NTM	$Ni_{0.8}Co_{0.2}$@NTM	$Ni_{0.7}Co_{0.3}$@NTM
Ni	4.18	4.51	1.61
Co	0.61	1.19	0.79
Co% in Ni/Co alloy	12.7%	20.8%	32.9%

Reference:

1. Kresse, G. and J. Furthmüller, *Efficiency of ab-initio total energy calculations for metals and semiconductors using a plane-wave basis set.* Computational Materials Science, 1996. **6**(1): p. 15-50.
2. Kresse, G. and D. Joubert, *From ultrasoft pseudopotentials to the projector augmented-wave method.* Physical Review B, 1999. **59**(3): p. 1758-1775.
3. Pack, J.D. and H.J. Monkhorst, *"Special points for Brillouin-zone integrations"---a reply.* Physical Review B, 1977. **16**(4): p. 1748-1749.
4. Voiry, D., et al., *Enhanced catalytic activity in strained chemically exfoliated WS2 nanosheets for hydrogen evolution.* Nature Materials, 2013. **12**: p. 850.
5. Zou, Y., et al., *Low temperature synthesis of single-phase Ti3AlC2 through reactive sintering Ti/Al/C powders.* Materials Science and Engineering a-Structural Materials Properties Microstructure and Processing, 2008. **473**(1-2): p. 90-95.
6. Ghidiu, M., et al., *Conductive two-dimensional titanium carbide 'clay' with high volumetric capacitance.* Nature, 2014. **516**(7529): p. 78-81.
7. Alhabeb, M., et al., *Guidelines for Synthesis and Processing of Two-Dimensional Titanium Carbide (Ti3C2TX MXene).* Chemistry of Materials, 2017. **29**(18): p. 7633-7644.
8. Zhao, L., et al., *Interdiffusion Reaction-Assisted Hybridization of Two-Dimensional Metal-Organic Frameworks and Ti3C2Tx Nanosheets for Electrocatalytic Oxygen Evolution.* ACS Nano, 2017. **11**(6): p. 5800-5807.
9. Liu, J., et al., *Hydrophobic, Flexible, and Lightweight MXene Foams for High-Performance Electromagnetic-Interference Shielding.* Advanced Materials, 2017. **29**(38): p. 1702367.
10. Ran, J., et al., *Ti3C2 MXene co-catalyst on metal sulfide photo-absorbers for enhanced visible-light photocatalytic hydrogen production.* Nat Commun, 2017. **8**: p. 13907.
11. Halim, J., et al., *X-ray photoelectron spectroscopy of select multi-layered transition metal carbides (MXenes).* Applied Surface Science, 2016. **362**: p. 406-417.
12. Ramanath, G., et al., *W deposition and titanium fluoride formation during WF6 reduction by Ti: Reaction path and mechanisms.* Journal of Applied Physics, 1999. **85**(3): p. 1961-1969.
13. Baltrusaitis, J., D.M. Cwiertny, and V.H. Grassian, *Adsorption of sulfur dioxide on hematite and goethite particle surfaces.* Physical Chemistry Chemical Physics, 2007. **9**(41): p. 5542-5554.

14. Hu, W., et al., *Deactivation mechanism of arsenic and resistance effect of SO42− on commercial catalysts for selective catalytic reduction of NOx with NH3.* Chemical Engineering Journal, 2016. **293**: p. 118-128.
